# Senescent Nephropathy: The New Renal Syndrome

**DOI:** 10.3390/healthcare5040081

**Published:** 2017-10-28

**Authors:** Florencia Aiello, Eliana P. Dueñas, Carlos G. Musso

**Affiliations:** 1 Human Physiology Department, Instituto Universitario del Hospital Italiano de Buenos Aires, Buenos Aires C1199ABB, Argentina; instituto.universitario@hospitalitaliano.org.ar; 2 Family Physician Department, Universidad del Valle, Valle del Cauca 760001, Colombia; eliana.711410002@ucaldas.edu.co; 3 Geriatric Internal Medicine Department, Universidad de Caldas, Manizales 170002, Colombia

**Keywords:** chronic kidney disease, ageing, senescence, frailty

## Abstract

Chronic kidney disease (CKD) is a condition characterized by progressive and irreversible deterioration of renal function due to the reduction of nephron mass for a period of at least three months. The prevalence of CKD is roughly 10% in the general population but increases with age, affecting more than one-third of people older than 65. Frailty is a condition usually found in elderly people, characterized by weakness, motility, and balance issues, with a declined ability to resist stressors leading to increased risks of adverse health outcomes including falls, fracture, hospitalization, institutionalization, disability, dependence, dementia, poor quality of life, and death. There is interdependence between CKD and normal ageing whereby CKD makes ageing more accelerated and pronounced (senescence), whereas senescence accelerates chronic nephropathy’s progression. Frailty status catalyzes this spiral, with renal and systemic consequences, phenomenon which can be named senescent nephropathy. In conclusion, senescent nephropathy is a new renal syndrome that should be taken into account, and we must try to handle its appearance and progression not only by applying nephron prevention measurements but also by diagnosis and treating frailty in the CKD population.

## 1. Introduction

Chronic kidney disease (CKD) is a condition characterized by progressive and irreversible deterioration of the renal function due to the reduction of nephron mass for a period of at least three months. In order to diagnose this disease, the presence of at least one of the following parameters is required: reduction in the glomerular filtration rate (GFR), altered urinalysis (persistent renal hematuria and/or albuminuria–proteinuria), and kidney structural abnormalities, detected by a biopsy or imaging [1].

Starting in one’s forties, the kidney experiences a progressive reduction in mass and a GFR decline at a rate of 1 mL/year. This phenomenon is associated with reduced renal cortical thickness and number of functioning nephrons, with histologic changes such as mild glomerulosclerosis, arteriosclerosis, tubular atrophy, and interstitial fibrosis [2]. Even though GFR is reduced in ageing and CKD, the magnitude of this reduction is the expected for age, with normal serum urea, creatinine, hematocrit, erythropoietin, parathyroid levels, as well as normal urinalyses and renal imaging in ageing, while at least one of these parameters is altered in CKD patients (Table 1) [3].

The prevalence of CKD is roughly 10% in the general population but increases with age, affecting more than one-third of people older than 65. Chronic kidney disease incidence rises steadily with age, making it at least 10 times more common in the oldest old (≥80 years of age) than in younger adults (18–50 years) [4]. Thus, a reduced GFR represents a significant mortality risk factor in the elderly when its value is lower than the expected for their age (≤40 mL/min/1.73 m²) [5].

Frailty is a condition usually found in elderly people, and is the opposite of fit status (where old people are freely ambulant and live independently at home). Moreover, frailty status is characterized by weakness, motility and balance issues, and a declined ability to resist stressors, leading to increased risks of adverse health outcomes including falls, fracture, hospitalization, institutionalization, disability, dependence, dementia, poor quality of life, and death [4,6]. These frailty complications explain the importance of trying to delay this process and its consequences [7].

In the present article we describe the interrelationship between CKD, ageing, and frailty, as well as the deteriorating spiral they install.

## 2. Ageing and Frailty Status

There is a considerable difference between normal ageing (successful ageing) and pathologic ageing (senescence) since the physiological changes of old age prevail in the former, while these changes appear earlier (accelerated ageing) or with more magnitude (disease) in the latter [3]. Additionally, senescent people characteristically have a reduced homeostatic capability, which makes their bodies frail [8]. Frailty is a dynamic process, but transition to a worse state is more common than to a better state, since frailty usually leads to a spiral of increasing decline and risk of disability, falls, and death [9].

The prevalence of frailty among community-dwelling people increases steadily with age, being 4–7% in old people, and 9–26% in very old people. Moreover, female gender and chronic diseases also increase the frailty phenotype prevalence [6,9].

Regarding frailty pathophysiology, this consists of a loss of complexity, with a reduced physiological reserve (<30%), altered multisystem regulation, and consequently a reduced homeostasis that leads to vulnerability, morbidity, and mortality. Chronic inflammation, immune activation, as well as musculoskeletal and endocrine alterations are involved in frailty development; all these processes are modulated by metabolic, genetic, epigenetic, and pathologic factors [10]. Regarding the ageing inflammatory process (“inflammaging”), it constitutes a complex response to internal and external influences mediated by an increase in pro-inflammatory cytokine levels. The transition from successful ageing to senescence could be related to a cytokine production deregulation with an excessive increase in tumor necrosis factor, interleukin-1, and interleukin-6 levels. Oxidative stress could be the first factor to contribute to “inflammaging,” not only through reactive oxygen species (ROS) production but through inducing the transcription of genes involved in the production of cytokines and inflammatory proteins [7]. Moreover, oxidative stress stimulates the inflammation factor, whereas inflammatory changes increase ROS production, creating a vicious circle that leads to a higher increase in the oxidative stress and inflammatory state [6,11,12].

Sarcopenia is the progressive and generalized loss of musculoskeletal mass and strength that occurs with advancing age, which increases the risk of adverse outcomes such as physical disability, poor quality of life, and death. Sarcopenia diagnosis is based on muscle mass assessment by body imaging techniques, bioimpedance analysis (lean body mass), muscle strength evaluated by handgrip strength, and clinical scores. However, frailty is more multifaceted than sarcopenia alone, since frailty goes beyond physical factors to encompass physiological and social dimensions. The frailty phenotype is often exacerbated by social isolation, depression and cognitive impairment, which reinforce the frailty status, establishing a vicious circle [9].

Some authors include sarcopenia as one of the diagnostic criteria for frailty phenotype. Frailty phenotype is usually accompanied by insulin resistance and insufficiently regulated inflammatory response, and consequently an important release of activating muscle breakdown cytokines (IL-6 and TNF-alfa) that induce sarcopenia and loss of muscle strength [4,9].

CKD is one of the diseases that can be involved in the onset and progression of sarcopenia by different mechanisms such as malnutrition, low vitamin D, hormonal decline, and metabolic acidosis [1,4,9].

## 3. Frailty Status Evaluation

Based on two different conceptual models of frailty, two main frailty assessment tools have emerged: the fragility phenotype coined by Fried et al. (2001), and the Fragility Index (FI) coined by Jones et al. (2004). However, other diagnostic criteria, such as FRAIL (Fatigue, Resistance, Ambulance, Illnesses, Loss of weight) and SHARE-FI (Frailty Instrument for Primary Care of the Survey of Health, Aging and Retirement in Europe) have been proposed [10,13,14].

The frailty phenotype incorporates disturbances across interrelated domains to identify old people who are at risk of disability, falls, institutionalization, hospitalization, and premature death. Fried et al. included the following domains: shrinking, weakness, poor endurance and energy, slowness, and low physical activity level. Those with three or more of the five domains are judged to be frail, while those with one or two domains are pre-frail, and those with none are deemed fit or robust elderly people [6,13]. Even though the frailty phenotype and comorbidity (the presence of two or more chronic diseases) are related terms, they should not be used interchangeably. The frailty phenotype causes disability independently of comorbidity due to weakness, poor endurance, and slowness. In this sense, the frailty phenotype without comorbidity was documented in 27% of the aged population, while comorbidity was observed in around 46% of this group [9].

The FI is based on a multidimensional geriatric assessment, which evaluates the accumulation of deficits, including disease, physical and cognitive alterations, and psychosocial risk factors. FI is a more sensitive predictor of adverse health effects. Rockwood et al. also designed a screening instrument based on clinical judgment: The Clinical Frailty Scale (CFS) indicates the different stages of frailty in a patient. CFS is a validated tool for diagnosing frailty, and consists of an evaluation of seven to nine items indicating the overall clinical status of an elderly person based on the evaluation of their state in the domains of mobility, energy, physical activity, function, and prognosis [15,16].

Similarly, physical performance can be assessed by applying several clinical tests such as short physical performance battery or timed get up and go tests, but gait speed, also often termed walking speed, seems to be the more recommended test, since it has been associated with survival in the elderly. Gait speed uses the instructions to walk at usual pace and from a standing start, and, assessing the seconds elapsed with a stopwatch, walking 15 feet (4.5 m). Gait speed slower than 0.6 m/s has been associated with poor clinical outcome, and a cutoff around 0.8 m/s has been proposed as a predictor of life expectancy [17].

Regarding the diagnosis of frailty in CKD patients, it could serve as a useful clinical instrument for summarizing the cumulative burden of physiological impairments in this group. Frailty phenotype was documented to be associated with a great risk of dialysis need or death after adjustment for concomitant chronic diseases [9,18].

Because geriatric patients with CKD are a heterogeneous group, they should be stratified as healthy, vulnerable, or fragile based on geriatric assessment and defining their functional status, psychosocial status, and support system at home, since these factors will impact on the prognosis. Thus the classification of the elderly into one of the following categories can be useful for making clinical decisions in this group [19]:

- Fit status: These patients are optimal candidates for dialysis and transplantation.
- Vulnerable (pre-frail): In these patients, geriatric assessment and intervention plans, such as rehabilitation, pain management, treatment of cognitive deficits and depression, management of polypharmacy, and/or prevention of falls, may slow the progression of the deteriorating factors and better the quality of life and the experience of dialysis.
- Frail: These patients are at risk of suboptimal dialysis and should be considered for a non-dialysis treatment plan (conservative treatment) or a short-term dialysis test.

Frailty must be evaluated in order to initiate adequate treatment in the CKD patient due to its important implication in morbimortality in this group [4].

It is of the utmost importance to examine frailty status in the ESRD population as they are at the highest risk among CKD patients [6]. Low levels of physical activity found in ESRD can tend to over-detect the frailty phenotype in this group, which the reliable tests mentioned above can help to avoid [9]. 

Since frail patients with ESRD are at high risk of negative health outcomes, it is worthwhile to identify frail patients with ESRD who may potentially benefit from interventions, such as a nutritional supplement or exercise program [6]. Although rehabilitation in these patients may be cost-effective and prevent institutionalization, these services are usually underutilized in this population [20,21,22].

## 4. Ageing, CKD, and “Senescent Nephropathy”

CKD is a highly prevalent disorder in people older than 65 years of age in Western societies. It is known that these patients have a reduced GFR due to the combination of ageing and chronic nephropathy damage [23]. CKD is a condition associated with malnutrition, chronic inflammation, increased oxidative stress, protein–energy wasting, acidemia, impaired hormonal changes, anemia, accumulation of advanced glycation end-products, insulin resistance, vessels calcification, reduction in bone mass, altered regulation of body methionine transmethylation reactions by the kidney, and low physical activity, all of which can directly and indirectly contribute to accelerate and worsen the aging process (senescence), and consequently to develop the frailty phenotype [4,6,9]. Furthermore, CKD was found to be significantly associated with depression, cognitive decline, functional limitation, and falls and their complications in the elderly [9,18]. Sarcopenia increases progressively, along with the loss of renal function in CKD patients, a phenomenon that seems to be primarily due to type II fiber atrophy influenced by altered protein turnover rates and mitochondrial depletion. Moreover, it has been shown that CKD progression decreases protein intake, especially when the GFR is below 25 mL/min/1.73 m², worsening patient’s sarcopenia [24,25]. CKD-associated muscle mass loss justifies the high prevalence of low grip strength and slow walking speed in this population, thus a low exercise capability is a powerful independent predictor of mortality in this population [9]. Although CKD itself is a predictor of adverse health outcomes, coexistence of CKD and frailty has been shown to further increase risks of falls, fractures, hospitalization, and mortality [4,6]. Additionally, CKD comorbidities (diabetes mellitus, etc.) are also associated with physical disability in the general population [9]. Sarcopenia and frailty prevalence increases with age as well as with kidney disease stage. Frailty prevalence is estimated as 14% in the elderly with non-dialytic CKD, and up to 40–73% in those who are on dialysis therapy [9,19].

The dialytic treatment seems to prolong longevity in the elderly compared to conservative treatment, but not in the sickest elderly patients. Mortality at 12 months after initiating dialysis exceeds 35% among patients over 70 years of age and exceeds 50% among patients over 80 years of age [9,20]. Some authors have reported a loss of independence in very old CKD patients who started dialysis treatment, observing that many patients suffer a deterioration in their functional state three months from the beginning of dialysis, with a negative impact on their daily activities [4,20]. A retrospective study suggested an association between frailty and increased GFR at the start of dialysis. This relationship can be explained by the fact that the signs and symptoms of end-stage renal disease are not specific and it is possible that the presentation of the fragility in these patients is assumed to be a symptom of uremia and in this way leads to an earlier onset of dialysis. In addition, it is considered that GFR may be overestimated in these patients and they expect dialysis to alleviate their fragility symptoms and have fewer activities that are limited by dialysis [18]. Thus, in order to determine the best time to initiate dialysis, it would be important to consider factors other than nephrological ones (GFR, electrolyte levels, etc.), and frailty status should also be included in this assessment [18].

There is an interdependence between CKD and ageing whereby CKD accelerates and worsens ageing (senescence), while senescence accelerates CKD progression, since frailty status is the common path that catalyzes this spiral of damage. Since this particular subgroup of CKD frail elderly patients suffers from a condition with different clinical complications, therapy needs, and overall prognosis compared to CKD fit elderly patients, we propose naming this clinical setting with a new term: “senescent nephropathy“. This name condenses the concept of the deleterious and progressive renal and systemic consequences of the negative interdependence between CKD and senescence, and consequently contributes to better individualizing this condition, promoting its prompt diagnosis and treatment. Thus, every CKD elderly patient should be evaluated in order to discard the presence of the frailty phenotype, applying any of the validated scores above described. If the presence of the frailty phenotype is documented, rehabilitation therapy should be added to the CKD treatment. In addition, this rehabilitation therapy should be in accordance with the frailty-inducing mechanism, for instance: if sarcopenia is detected as the patient’s frailty-inducing factor, a normal diet (instead of a low-protein diet) and muscle exercise should be prescribed. Moreover, conventional CKD therapeutic measures, such as antiproteinuric agents, immunosuppressants, dialysis, etc. should be implemented more carefully (lower doses and tighter control) since they can be less tolerated in this group (Table 2). 

## 5. Conclusions

Senescent nephropathy is a new renal syndrome that should be taken into account, and its appearance and progression should be treated not only by applying nephropreventive measures but also by diagnosing and treating frailty status in CKD patients.

## Figures and Tables

**Table 1:** Clinical guide for distinguishing an aged kidney from a chronically damaged one.

	Renal Ageing	Stage III–CKD	SN
GFR	low (expected value for age)	low (any value)	low (any value)
Serum urea	normal	high	high
Serum creatinine	normal	high	high
Hematocrit	normal	normal/low	normal/low
Parathyroid hormone	normal	elevated	elevated
Urinalysis	normal	normal/altered	normal/altered
Renal image	normal	normal/abnormal	normal/abnormal
Clinical functional status	fit / frail	fit	frail

GFR: glomerular filtration rate; CKD: chronic kidney disease; SN: senescent nephropathy.

**Table 2:** Differences between chronic kidney disease (CKD) in fit elderly and senescent nephropathy (SN) patients.

	CKD	SN
CKD diagnosis	positive	positive
Frailty phenotype score	negative	positive
Treatment	corresponding CKD therapy	corresponding CKD therapy adjusted to frailty status + frail rehabilitation & home assistance
CKD follow-up	Standard control rate	tighter control rate
CKD prognosis	standard	worse
